# Editorial: Advancements in mental health services: elucidated promising new paths along which to seek answers to improve our mental health systems

**DOI:** 10.3389/frhs.2025.1758517

**Published:** 2026-01-30

**Authors:** Carolyn S. Dewa

**Affiliations:** Department of Psychiatry and Behavioral Sciences, School of Medicine, University of California, Davis, CA, United States

**Keywords:** acceptability, access, mental health services, participatory action research, quality of care

This *Frontiers in Health Services* research topic sought studies offering new insights into novel developments and current challenges in the field of mental health services. The included studies highlight the global struggle to make services more accessible, a challenge exacerbated by the COVID-19 pandemic (1). Levesque et al. (2) conceptualized access to healthcare in terms of five dimensions: (1) approachability, (2) acceptability, (3) availability, (4) affordability, and (5) appropriateness. Acceptability and appropriateness underlie many of the research questions these studies addressed.

## Considering the acceptability of services

Levesque et al. (2) defined acceptability as involving cultural and social factors that affect a prospective service user's perception of the appropriateness of seeking services. This aspect of access recognizes the importance of patient-centered care. Patient-centered care ensures that care is “respectful and responsive to individual patient preferences, needs and values” (3) [SIC]. The Institute of Medicine (IOM) suggests that this approach is critical to providing quality care (3).

Reynolds et al.'s pilot study of the CONNECT program focused on increasing the accessibility of mental health support services for Canadian seniors through telephone-based groups. Their research explored whether this mode of support would be acceptable to this group using mixed methods; the evidence suggests it is.

Using secondary data, Taube looked at the effects of introducing psychiatric outpatient centers in Latvia. As hypothesized, the study observed a decrease in inpatient length of stay and an increase in outpatient use. However, there was no decrease in inpatient admissions. Furthermore, there were differences among subgroups. The author reported that, while increases in outpatient clinic use were observed for those with depression, this trend did not extend to those with psychotic and/or organic psychiatric disorders. Thus, as she points out, it will be important to investigate the underlying causes of these differences.

Using qualitative methods, Cheng et al. investigated ways to make research involvement more acceptable to youth. Their findings highlight: (1) the need to make research participation meaningful and (2) the importance of using multimodal data collection methods. They also noted that the youth who agree to join participatory research studies are not necessarily the program service users. Thus, the information collected about the acceptability of services may not necessarily reflect the preferences of the target population.

## Considering the appropriateness of services

Levesque et al. (2) conceptualized appropriateness as encompassing the match between need and treatment, in addition to the quality of services, which includes factors of integration and continuity. In their pilot study examining telephone-based groups for seniors, Reynolds et al. reported that the program was effective in impacting depression, providing emotional support, and improving mental health literacy. However, they also noted that future research is needed to understand the generalizability of these promising results and their acceptability to racially/ethnically, socioeconomically, and gender-diverse populations.

Alemu et al. used survey data to investigate satisfaction with outpatient clinic services in Ethiopia. The authors found that approximately 75% of service user respondents were satisfied with the services. Focusing on the 24% of respondents who reported low satisfaction, they explored the significant characteristics of this group and found that they were more likely to live in urban areas, have poor self-reported health, experience episodic illness and relapse, and poorly adhere to medication regimes. These findings suggest the need for further research that uses qualitative methods to investigate the experiences of these groups. The authors also noted that service users who were experiencing severe illness at the time of the study and those who did not attend their follow-up visits were not surveyed. The lack of their responses could have affected the survey results, as their perspectives could be another source of information about access barriers.

Wang et al. studied an integrated treatment model introduced in a psychiatric hospital and emergency setting in China. The purpose of the model was to respond to critical physical healthcare needs within the psychiatric inpatient setting. The service was created to recognize that there are critical physical health complications associated with psychiatric disorders and symptoms. This model simplifies care delivery, as the IOM (3) asserts is needed to improve the quality of care. The authors acknowledge that further research is needed to examine the effectiveness of the model along with the patient experience.

In their systematic literature review, Barr et al. examined evidence of the impact of animal ownership, pet attachment, and interaction on common mental disorders. The study sought to understand whether pet ownership could supplement mental health support when it is limited or not readily available. The authors found evidence of an association between pet ownership and lower depression scores. However, the pattern was unclear for those with anxiety. This finding underscores the importance of identifying the specific mental health condition. Their study also pointed to gaps in the literature related to the often-used cross-sectional design and emphasized the need for longitudinal studies.

Obegu et al. used qualitative methods to examine the perspectives of family caregivers of individuals with severe mental disorders. Their findings highlight the complexity of the mental health system and the need for help to navigate it. The interviewed caregivers expressed the need for guidance, knowledge, and more providers. These results offer insight into future areas of research to understand the preferences of caregivers – how they would like to be supported and what characteristics the support should have.

This research topic featured studies that focused on improving access to care. Among them, the novel approaches ranged from using a 148-year-old technology to employing non-human companions. Other contributions expanded the concept of “specialty” to encompass non-specialty care. These articles demonstrate the importance of understanding the user experience and the power of mixed methods. Together, they elucidate promising new paths to improve our mental health systems.
